# Foam Rolling as a Recovery Tool Following Eccentric Exercise: Potential Mechanisms Underpinning Changes in Jump Performance

**DOI:** 10.3389/fphys.2019.00768

**Published:** 2019-06-26

**Authors:** Eric J. Drinkwater, Christopher Latella, Christopher Wilsmore, Stephen P. Bird, Melissa Skein

**Affiliations:** ^1^Centre for Sport Research, School of Exercise and Nutrition Sciences, Deakin University, Geelong, VIC, Australia; ^2^Centre for Exercise and Sports Science Research (CESSR), School of Health and Medical Sciences, Edith Cowan University, Joondalup, WA, Australia; ^3^School of Exercise Science, Sport and Health, Charles Sturt University, Bathurst, NSW, Australia; ^4^Medical and Exercise Science, School of Medicine, University of Wollongong, Wollongong, NSW, Australia

**Keywords:** self-massage, myofascial release, resistance training, power, delayed onset muscle soreness, muscle damage

## Abstract

**Purpose:**

Recovery from exercise-induced muscle damage (EIMD) is paramount in sports performance. Foam rolling (FR) has been suggested to improve acute performance; however, the ability to facilitate recovery from eccentric (ECC) exercise remains unclear.

**Methods:**

Eleven males undertook 6 × 25 ECC knee extensions to induce muscular damage. Immediately, 24, 48, and 72 h post-training countermovement jump (CMJ), maximal voluntary isometric contraction (MVIC), pressure-pain threshold (PPT), knee flexion range of motion (ROM), and mid-thigh circumference (MTC) were assessed. Neurophysiological measures included voluntary activation (VA), peak twitch torque (PTT), time to peak twitch (PTT_time_), and rate of twitch torque development (RTD). Participants then spent 15 min FR prior to each time point or control (CON). Repeated measures analysis of variance (ANOVA) and standardized effect sizes (Hedges’ *g*) ± 95% confidence intervals (95% CI) were used to compare FR and CON.

**Results:**

CMJ was greater for FR compared to CON (*P* = 0.030) at 72 h (8.6%, *P* = 0.004) with moderate effects observed at 48 and 72 h (*g* = 0.54–0.66). PPT was greater with FR (*P* = 0.018) at 48 h only (23.7%, *P* = 0.013), with moderate to large effects noted at all-time points (*g* = 0.55–0.98). No significant differences were reported for MVIC (*P* = 0.777, -5.1 to 4.2%), ROM (*P* = 0.432, 1.6–3.5%), VA (*P* = 0.050, 3.6–26.2%), PTT (*P* = 0.302, -3.9 to 9.9%), PTT_time_ (*P* = 0.702, -24.4 to 23.5%), RTD (*P* = 0.864, -16.0 to -1.0%), or MTC (*P* = 0.409, -0.5 to -0.1%) between conditions.

**Conclusion:**

FR appears to improve jump performance in the later stages of recovery following ECC exercise. This may be in part due to improved pain tolerance; however, mechanical and neurophysiological are not modulated with FR.

## Introduction

Following training and competition, reductions in neuromuscular performance occur at least in part, due to acute fatigue and longer-lasting EIMD. EIMD is common following intense exercise, especially when repeated ECC contractions are performed. Consequently, ECC exercise can impair neuromuscular function for prolonged periods of time due to muscle soreness and pain, structural perturbations, and inflammation (Kouzaki et al., 2016). In sport, the spatiality of training sessions and/or competition are often more frequent than the ideal recovery period, leading to sub-optimal performance, burnout, and injury (Lehmann et al., 1999; Kellman, 2010). Therefore, several, often concurrent techniques are employed in an attempt to facilitate recovery. For example, contrast- or cryo-therapy, stretching, massage, light exercise, and FR are commonly used, however, the supporting evidence and the understanding of potential underlying mechanisms are largely inconclusive (Luttrell and Halliwill, 2015).

It is well established that fatigue occurs via a combination of central (neural) and peripheral (muscular) mechanisms (Enoka and Duchateu, 2016). For instance, a reduction in central drive to the muscle (i.e., VA) has been shown following sustained isometric tasks (Gandevia et al., 1996; Taylor and Gandevia, 2008) and in particular, the days following ECC exercise (Behm et al., 2001; Prasartwuth et al., 2005). Other evoked contractile properties (e.g., PTT and RTD) may also be compromised under fatigue. Furthermore, neuromuscular performance can also be impaired by various physiological processes at the muscular level including metabolic perturbations and mechanical stress (Allen et al., 2008; McKenna et al., 2008). In the days following repeated ECC contractions, pro-inflammatory responses induce swelling and increase pain sensitivity, likely due to structural damage of the myofibrils, cellular matrix, and connective tissue (Proske and Allen, 2005; Kanda et al., 2013). Thus, a reduction in neuromuscular function may severely impact performance and increase the likelihood of injury, especially following muscle damaging ECC exercise.

In recent years FR, a form of self-massage, has gained popularity in sports science settings. However, despite continued scientific enquiry the effectiveness of FR to improve functional performance and recovery, and the underlying mechanisms that may be responsible remain somewhat unclear. Previous literature has sought to investigate the various mechanical, tissue, perceptual, and functional responses when FR is employed (Cheatham et al., 2015; Schroeder and Best, 2015; Behara and Jacobson, 2017; Schroeder et al., 2018). Specifically, an acute decrease in tissue stiffness (Krause et al., 2017) improved joint ROM (MacDonald et al., 2013, 2014; Cheatham et al., 2015; Schroeder and Best, 2015; Kalichman and David, 2017; Su et al., 2017; Smith et al., 2018), reduced soreness (MacDonald et al., 2013; Beardsley and Skarabot, 2015; Paz et al., 2017), and reduced perceptions of pain and fatigue (Rey et al., 2017; Richman et al., 2018) have been reported. However, the effects of FR on maximal strength and power expression are mixed (MacDonald et al., 2013; Halperin et al., 2014; Jones et al., 2015; Richman et al., 2018; Smith et al., 2018). In particular, several studies have investigated the effects of FR following exercise in the lower limbs (MacDonald et al., 2013; Behara and Jacobson, 2017; Cavanaugh et al., 2017a) with authors reporting improvements in either ROM, jump height, power, sprint performance, or change of direction (MacDonald et al., 2014; Cheatham et al., 2015; Pearcey et al., 2015; Schroeder and Best, 2015; Freiwald et al., 2016; D’Amico and Gillis, 2017; Rey et al., 2017; Richman et al., 2018). Recent evidence has also suggested that FR may benefit functional outcomes during the recovery period (Fleckenstein et al., 2017) despite no changes in tissue properties (Schroeder et al., 2018); however, specific ECC exercise studies are limited (Pearcey et al., 2015; Romero-Moraleda et al., 2017). Additionally, it is unclear if neurophysiological mechanisms (i.e., VA or PTT) contribute to the performance improvements (i.e., jump performance) often observed following FR.

The aim of this study was to investigate the effects of acute FR on the functional, mechanical, and subjective outcomes, and neurophysiological mechanisms following a single bout of ECC exercise. Specifically, we aimed to quantify these responses during the fatigue and recovery period, up to 72 h post-exercise. Based on the previous evidence, we hypothesized that: (1) the recovery of performance variables (MVIC and/or CMJ) will be facilitated with a FR intervention and (2) improved neural, mechanical, and subjective outcomes will accompany an improvement in performance. The results are expected to provide evidence regarding the efficacy of FR as a tool to improve functional recovery and elucidate the potential underpinning neurophysiological mechanisms responsible. These findings will be particularly important for athletes who have consecutive bouts of training and competition resulting in muscle damage with minimal inter-session recovery periods.

## Materials and Methods

### Experimental Approach to the Problem

Following an initial familiarization session, each participant was involved in two identical ECC protocols with 3 weeks between sessions in a randomized, counter-balanced cross-over design. The two experimental conditions consisted of the ECC exercise followed by either: (1) quiet sitting for 15 min rest following exercise and before each testing point; CON or (2) completed 15 min FR immediately post-training and before each testing point and at 24, 48, and 72 h at the same time of day for each participant across both conditions. The order of testing was as follows: MTC, ROM, CMJ, and then MVIC followed by electrical stimulation.

### Participants

Eleven healthy young males (age: 24.0 ± 0.7 years, height: 180.0 ± 7.0 cm, body mass: 82.0 ± 7.0 kg) with at least 2 years of regular (≥2 days per week) general resistance training experience and no report of lower extremity injuries within the last 6 months volunteered for this study. Participants were asked to abstain from food and caffeine 3 h prior to testing, and physical activity and alcohol 24 h prior to testing and during recovery. Participants were informed of the study requirements and written consent was obtained prior to testing. This study was approved by the Charles Sturt University Human Research Ethics Committee.

### Eccentric Exercise Protocol

The protocol designed to elicit muscle damage involved the participant seated upright on an isokinetic dynamometer (HUMAC NORM, CSMi Medical Solutions, MA, United States) with the knee and hip positioned at 90° of flexion. The participant was secured with a harness and the leg secured to the lever arm with a strap placed at the ankle 1 cm above the lateral malleolus. The axis of rotation of the dynamometer was aligned with the lateral epicondyle of the right femur. During all contractions the participant placed the arms across the chest. The protocol involved 150 ECC contractions segmented into 6 sets of 25 minimally resisted knee extensions and maximally resisted ECC flexion of the right knee (30**°** s^-1^ extension and 120**°** s^-1^ flexion). Each set was separated by 60 s of passive recovery. Strong verbal encouragement was provided to the participant throughout each set to ensure maximal effort. The ECC protocol was centered on eliciting DOMS in the quadriceps (agonist), however, due to the biomechanical movement employed, resultant effects on the antagonist and synergist muscles were also likely.

### Foam Rolling

The FR intervention specifically targeted five lower extremity areas (3 min per area) of the right leg as previously described by Pearcey et al. (2015). The participant consistently placed as much body mass as bearable onto the foam roller (HART Sport Foam Roller, 30 cm × 15 cm, Virginia, QLD, Australia) and was instructed to roll their body weight along the roller as evenly as possible at a rate of one rolling motion per second. The description and order of the areas targeted include: (i) quadriceps: the participant commenced in a prone position with one leg over the other. The roller moved from the anterior superior iliac spine to the patellar tendon with the participant using elbows to guide the movement, (ii) adductors: the participant commenced in a prone position with the hip positioned at 90° and externally rotated. The roller moved from the proximal portion of the adductor group (inferior to the inguinal area) to the medial condyle with a consistent shifting of body weight, (iii) iliotibial band: the participant commenced in a side lying position with the placement of the free leg anterior to the supported leg and rolled back and forth from the greater trochanter to the lateral condyle with the free foot controlling movement, (iv) gluteals: the participant commenced with one foot crossed over the opposite knee in a figure-four configuration while supporting body weight on the one hand. Utilizing the support hand, the participant rolled from the posterior portion of the iliac crest to the gluteal fold, and (v) hamstrings: the participant commenced with one foot crossed over the other and body weight supported by the hands, posterior to the body and the participant rolled from the gluteal fold to the popliteal fossa. Standardization of the positioning for each participant was provided during the familiarization and monitored throughout the intervention by the research team.

### Mid-Thigh Circumference

Mid-thigh circumference was assessed with a steel tape measure (MURATEC-KDS, F10-02, Kyoto, Japan) with the participant in the anatomical position. Girth measurements were recorded from the right thigh perpendicular to the long axis of the thigh, midway between the trochanterion and tibiale laterale. Results were recorded to the nearest millimeter and the mean was recorded from three consecutive measurements.

### Pressure-Pain Threshold

Pressure-pain threshold was assessed over the right rectus femoris. The participant was seated upright on a physiotherapy table with the hip and knee at 90° of flexion and popliteal fossa flush with the edge of the padded table. Following identification of the muscle belly of the rectus femoris, an algometer (Wagner Instruments, FDIX-RS232 Force One, Greenwich, CT, United States) was placed over the belly of the muscle with a downward pressure gradation of 1 kg cm^2^ s^-1^ until the participant acknowledged the initial point of shift in sensation from “pressure” to “pain.”

### Range of Motion

Knee flexion ROM of the right leg was assessed utilizing a modified Ely’s test and mechanical goniometer (JAMAR, Jackson, MI, United States) (Peeler and Anderson, 2008). Previous research suggests that Ely’s test demonstrates moderate reliability (Peeler and Anderson, 2008). The participant was placed in a prone position on the physiotherapy table and the axis of rotation of the goniometer was fixed to the tibiale laterale. The stationary arm was fixed to the trochanterion and the movement arm was rotated against the lateral malleolus. The movement of the limb through its ROM was controlled by the investigator’s even pressure placed against the participant’s ankle at a rate of approximately 5° s^-1^ and measurement was taken when the participant acknowledged the elicitation of pain (Pyne et al., 2012). The procedure was completed three times with the greatest ROM recorded.

### Countermovement Jump

The participant then completed a 5 min cycling warm-up at 60 rpm with a 2-kp resistance (Monark 828E, Monark Exercise AB, Varberg, Sweden). Following the warm-up, CMJ height was assessed. The participant completed five CMJs on a 100 × 80 cm contact mat (AXON Jump T, Kinematics Sports Test System, Version 2.01, Buenos Aires, Argentina) separated by 60 s recovery. Each jump consisted of the participant standing straight, feet shoulder-width apart with hands fixed on hips; the body then dropped to a self-selected depth and immediately followed by the highest jump possible. CMJ height was calculated using the flight time. The mean jump height was calculated from the five CMJs recorded.

### Isometric Voluntary Torque

Maximal voluntary isometric contraction was assessed using the right knee extensors conducted with the same participant set up as the ECC exercise protocol. The participant completed three MVICs of the right knee extensors (90^o^ of flexion) for 5 s duration (2 s ramp up, 3 s maximal effort), with 60 s recovery between each contraction to avoid the effects of fatigue. The best of the three trials was recorded as the MVIC.

### Evoked Responses

An additional three MVICs were superimposed with a constant current electrical stimulus when a steady plateau in peak torque was achieved. A potentiated twitch was also evoked 3–5 s after the contraction when the muscle was at rest. Electrical stimuli to knee extensors were delivered using 1.5 cm lead electrodes (Nicolet, Cardinal Health, Madison, WI, United States) placed over the femoral nerve on the thigh 1.5 cm inferior to the inguinal fold. The current was delivered via a stimulator (Digitimer Ltd., Welwyn Garden City, Hertfordshire, United Kingdom) using single square-wave pulse with a width of 200 μs, linked to a terminal block and a signal acquisition system (PXI1024; National Instruments, Austin, TX, United States). The electrical current was increased incrementally until a plateau in the PTT was achieved, and then increased by a further 10% to ensure supra-maximal stimulation. VA levels were calculated using the twitch interpolation technique and the formula 1 - $\left(\frac{\mathrm{superimposed}\_\mathrm{twitch}}{\mathrm{potentiated}\_\mathrm{twitch}}\right)$ × 100 (Todd et al., 2003). The maximal twitch was determined as the difference in peak voluntary torque in the 50 ms prior to the delivery of the stimulus and the peak evoked torque value from stimulation. The RTD was calculated as the time elapsed to reach PTT.

Mean torque–time curves from the potentiated evoked resting twitch determined: (1) peak potentiated twitch torque (PTT; highest evoked torque obtained); (2) time to peak potentiated twitch torque (PTT_time_; time between the onset of the potentiated twitch and the PTT); and (3) RTD. These procedures were performed using MatLabTM Software (R2009b 7.9.0.529, The Mathworks Inc., Natick, MA, United States).

### Statistical Analysis

Differences in the mean changes between the interventions (FR and CON) were determined for each outcome variable using a two-way repeated measures of analysis of variance (ANOVA). Where significance was detected a *post hoc* paired samples *t-*test was conducted to examine differences between conditions at each individual time point. Significance was set at *P* < 0.05. Additionally, effect sizes were calculated using Hedge’s *g* and expressed using the following criteria: trivial <0.2, small 0.2–0.49, moderate 0.5–0.79, and large >0.8. Only results with a moderate or large effect were reported. Precision of mean differences was expressed with the 95% confidence interval (95% CI), which defines the range representing the uncertainty in the true value of the (unknown) population mean. All effect size calculations were performed in Excel (version 2013; Microsoft Corporation, Redmond, WA, United States) and ANOVAs were performed using SPSS (version 25; IBM Statistics). To display the 95% confidence interval of the effect sizes, results are displayed graphically as the mean, upper and lower 95% confidence limits.

## Results

**FIGURE 1:**
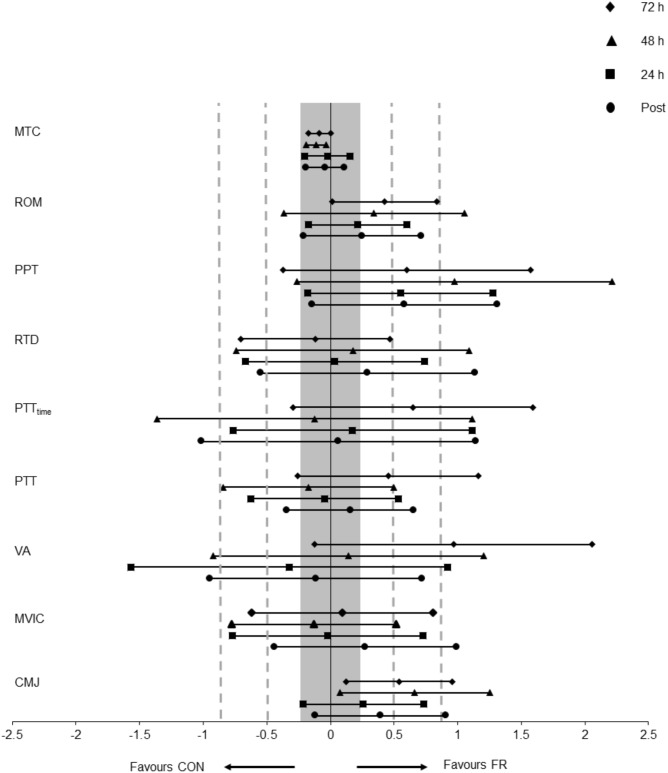
Displays the mean effect size (Hedges’s g) and 95% CI for each outcome variable; CMJ, MVIC, VA, PTT, PTT_time_, RTD, MTC, PPT, and Ely’s test for ROM for each condition (FR or CON) across all time points.

The values for each outcome measure are displayed in Table 1 and the effect sizes in Figure 1.

**Table 1:** The effect of the exercise protocol on each outcome variable: CMJ, MVIC, VA, PTT, PTT_time_, RTD, MTC, PPT, and Ely’s test for ROM for each condition (FR or CON) across all time points.

Variable	Time	CON (mean ± SD)	FR (mean ± SD)	CON Δ	FR Δ	Effect size (*g*, 95% CI)	Interaction	Condition	Time
CMJ	Pre	29.5 ± 4.6	29.4 ± 5.0				*F*_4,40_ = 2.994, *P* = 0.030^∗^	*F*_1,10_ = 4.640, *P* = 0.057	*F*_4,40_ = 4.618, *P* = 0.034^∗^
(cm)	Post	26.2 ± 4.1	27.8 ± 4.1	-3.4	-1.6	*0.39* (-0.12, 0.91)			
	24	27.2 ± 4.6	28.2 ± 4.8	-2.3	-1.1	0.26 (-0.22, 0.73)			
	48	27.7 ± 4.8	30.8 ± 5.3	-1.8	1.5	0.66 (0.07, 1.25)^#^			
	72	28.4 ± 4.4	30.7 ± 5.0	-1.1	1.4	0.54 (0.12, 0.96)^#^			

MVIC	Pre	139 ± 31	135 ± 33				*F*_4,40_ = 0.443, *P* = 0.777	*F*_1,10_ = 0.029, *P* = 0.869	*F*_4,40_ = 3.872, *P* = 0.009^∗^
(Nm)	Post	115 ± 34	122 ± 38	-23.8	-14.2	0.28 (-0.44, 0.99)			
	24	117 ± 42	113 ± 40	-22.0	-22.8	-0.02 (-0.77, 0.73)			
	48	129 ± 35	121 ± 42	-9.8	-14.4	-0.13 (-0,78, 0.51)			
	72	130 ± 36	130 ± 41	-8.7	-5.50	0.09 (-0.62, 0.80)			

VA	Pre	82.8 ± 17	74.3 ± 19				*F*_4,36_ = 2.627, *P* = 0.050	*F*_1,9_ = 0.730, *P* = 0.415	*F*_4,36_ = 1.572, *P* = 0.203
(%)	Post	83.3 ± 14	72.7 ± 25	0.5	-1.6	-0.11 (-0.94, 0.72)			
	24	71.9 ± 21	54.0 ± 46	-10.9	-20.3	-0.32 (-1.57, 0.92)			
	48	77.2 ± 19	71.2 ± 17	-5.6	-3.1	0.14 (-0.92, 1.20)			
	72	65.7 ± 27	77.0 ± 15	-17.1	2.7	0.97 (-0.12, 2.06)^#^			

PTT	Pre	64.2 ± 21	63.8 ± 17				*F*_4,40_ = 1.259, *P* = 0.302	*F*_1,10_ = 0.087, *P* = 0.773	*F*_4,40_ = 4.292, *P* = 0.031^∗^
(Nm)	Post	51.6 ± 21	54.1 ± 15	-12.6	-9.6	0.16 (-0.34, 0.66)			
	24	56.8 ± 15	55.6 ± 15	-7.4	-8.2	-0.05 (-0.62, 0.53)			
	48	65.1 ± 11	62.0 ± 13	0.9	-1.8	-0.17 (-0.84, 0.50)			
	72	55.1 ± 23	62.9 ± 11	-9.1	-0.8	0.45 (-0.26, 1.16)			

PTT_time_	Pre	174 ± 76	147.4 ± 43				*F*_4,40_ = 0.547, *P* = 0.702	*F*_1,10_ = 1.788, *P* = 0.211	*F*_4,40_ = 0.884, *P* = 0.482
(ms)	Post	160 ± 51	137 ± 72	-14.7	-10.6	0.07 (-1.01, 1.14)			
	24	179 ± 76	163 ± 74	4.3	16.1	0.17 (-0.76, 1.11)			
	48	186 ± 73	151 ± 58	12.0	4.1	-0.12 (-1.36, 1.11)			
	72	137 ± 62	149 ± 54	-37.4	1.4	0.64 (-0.29, 1.59)^#^			

RTD	Pre	460 ± 277	494 ± 241				*F*_4,40_ = 0.319, *P* = 0.864	*F*_1,10_ = 1.605, *P* = 0.234	*F*_4,40_ = 0.512, *P* = 0.727
(Nm.s^-1^)	Post	381 ± 201	484 ± 235	-79.0	-9.6	0.29 (-0.55, 1.14)			
	24	386 ± 229	428 ± 255	-74.7	-65.9	0.04 (-0.67, 0.74)			
	48	406 ± 164	479 ± 216	-54.1	-14.8	0.18 (-0.74, 1.09)			
	72	453 ± 229	460 ± 143	-7.1	-33.6	-0.12 (-0.71, 0.47)			

PPT	Pre	9.0 ± 2.0	7.9 ± 2.1				*F*_4,40_ = 3.372, *P* = 0.018^∗^	*F*_1,10_ = 0.026, *P* = 0.875	*F*_4,40_ = 5.153, *P* = 0.002^∗^
(kg^.^cm^2^)	Post	7.7 ± 2.5	6.8 ± 2.5	-1.3	-1.1	0.58 (-0.14, 1.31)^#^			
	24	6.8 ± 2.1	6.9 ± 2.8	-2.2	-1.0	0.55 (-0.18, 1.28)^#^			
	48	6.2 ± 2.1	7.5 ± 2.7	-2.8	-0.4	0.98 (-0.26, 2.21)^#^			
	72	8.0 ± 2.4	8.1 ± 2.7	-1.0	0.2	0.60 (-0.37, 1.57)^#^			

ROM	Pre	140 ± 7	141 ± 8				*F*_4,40_ = 0.881, *P* = 0.432	*F*_1,10_ = 6.744, *P* = 0.027^∗^	*F*_4,40_ = 1.869, *P* = 0.197
(°)	Post	136 ± 12	141 ± 19	-3.4	-0.3	0.25 (-0.21, 0.71)			
	24	136 ± 13	140 ± 11	-3.5	-1.4	0.22 (-0.17, 0.60)			
	48	137 ± 16	142 ± 10	-2.5	1.2	0.34 (-0.37, 1.05)			
	72	139 ± 15	146 ± 14	-0.5	4.5	0.42 (0.01, 0.84)			

MTC	Pre	54.8 ± 2.7	54.9 ± 3.0				*F*_4,40_ = 0.940, *P* = 0.409	*F*_1,10_ = 0.013, *P* = 0.911	*F*_4,40_ = 19.802, *P <* 0.001^∗^
(cm)	Post	55.6 ± 2.6	55.6 ± 2.9	0.8	0.7	-0.04 (-0.19, 0.11)			
	24	55.4 ± 2.7	55.5 ± 2.8	0.6	0.5	-0.02 (-0.20, 0.15)			
	48	55.4 ± 2.7	55.2 ± 2.9	0.6	0.2	-0.11 (-0.19, -0.03)			
	72	55.0 ± 2.8	54.9 ± 2.9	0.2	0.0	-0.08 (-0.17, 0.00)			


*^∗^Indicates significant difference between groups (*P* < 0.05), while ^#^ indicates and effect (Hedge’s g). All values are presented as Mean ± SD.*

### Neuromuscular Variables

**FIGURE 2:**
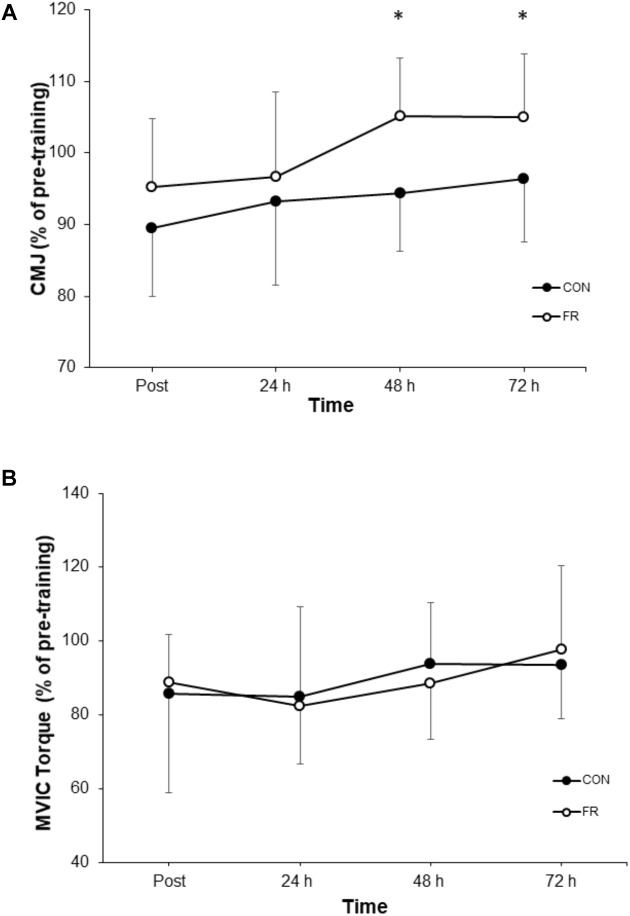
Shows the percentage change (±SD) from pre-training for **(A)** CMJ, and **(B)** MVIC between FR and CON. ^∗^Indicates significant difference between groups (*P* < 0.05).

A repeated measures ANOVA demonstrated a significant interaction (*P* = 0.030) and main effect of time (*P* = 0.034) for CMJ height between FR and CON. *Post hoc* analyses revealed that the recovery of CMJ height was greater for FR at 72 h (*P* = 0.004), compared to CON (Figure 2A and Table 1). Effect size analysis suggests a moderate effect for CMJ with FR 48 (*g* = 0.66) and 72 h (*g* = 0.54) compared to CON, respectively (Figure 1). No significant interaction was observed for MVIC (*P* = 0.777) between FR and CON (Table 1). Additionally, effect sizes were mostly trivial to small for FR on MVIC in comparison to CON across all time points (*g* = -0.13 to 0.28) (Figure 1, 2B).

### Mechanical Variables

**FIGURE 3:**
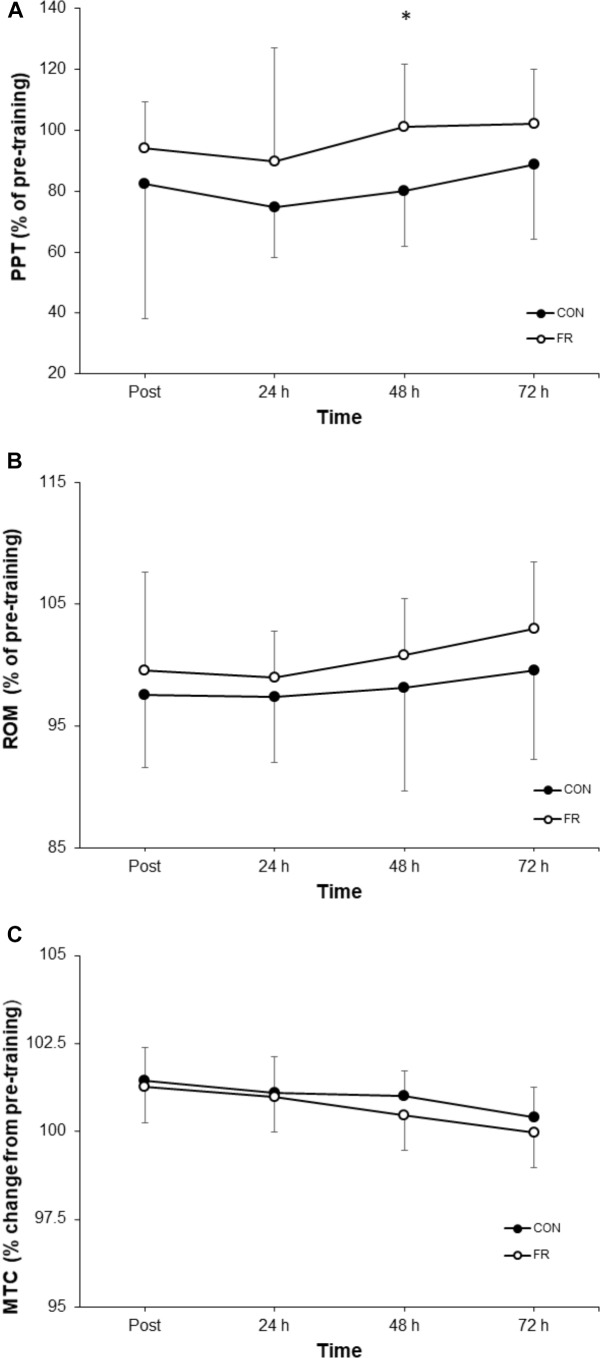
Shows the percentage change (±SD) from pre-training for **(A)** PPT, **(B)** ROM and **(C)** MTC between FR and CON. ^∗^Indicates significant difference between groups (*P* < 0.05).

A repeated measures ANOVA demonstrated a significant interaction (*P* = 0.018) and main effect of time (*P* = 0.002) for PPT between FR and CON. *Post hoc* analyses revealed that PPT was greater for FR at 48 h (*P* = 0.013) compared to CON (Table 1). Effect size analysis demonstrated a moderate effect for PPT with FR immediately post-training (*g* = 0.58) at 24 (*g* = 0.55), 48 (*g* = 0.98), and 72 h (*g* = 0.60) when compared to CON; however, these results did not reach statistical significance (Figure 1, 3A and Table 1).

No significant interaction was observed for ROM (P = 0.881) between FR and CON (Figure 1, 3B). No interaction was observed for MTC (P = 0.940) between FR and CON (Figure 1, 3C).

### Neural Variables

**FIGURE 4:**
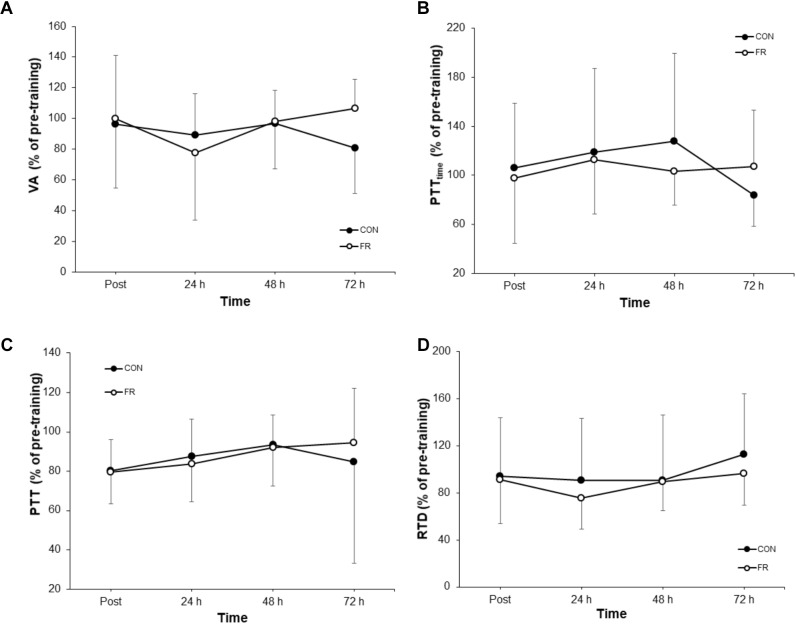
Shows the percentage change (±SD) from pre-training for **(A)** VA, **(B)** PTT_time_, **(C)** PTT and **(D)** RTD between FR and CON.

No significant interactions were observed for VA, PTT, PTT_time_ or RTD (Table 1). Additionally, there were no substantial effects of FR for VA (Figure 4A), PTT_time_ (Figure 4B) and PTT (Figure 4C) and RTD (Figure 4D) at most if not all time points, however a large effect size was observed for VA (*g* = 0.97) at 72 h.

## Discussion

Muscle damaging ECC exercise can impair performance for several days or longer. Due to the known debilitating effects on performance the aim of this study was to investigate if FR can improve functional recovery and in addition identify the potential underlying mechanisms that may contribute to this response. Specifically, we investigated neuromuscular (MVIC and CMJ), neural (VA, RTD, PTT, PTT_time_) and mechanical (ROM, MTC, PPT) outcomes in the lower limbs. The results showed significant improvements in CMJ at 72 h, with small to moderate effects observed at post-training and 48 h. Pain tolerance also increased at 48 h, with effects also observed at post-training, 24 and 72 h, respectively. No clear significant differences were observed during the recovery period for all other variables. Collectively, the results suggest that FR improves jump performance during recovery which may be at least partly mediated by and increased quadriceps pain tolerance, despite no improvement in maximal isometric force. FR may be an advantageous tool to aide recovery following muscle damaging ECC exercise, however it appears unlikely that neurophysiological mechanisms contribute to performance improvements.

Performance in the CMJ was greater at 72 h for FR compared to CON, with a moderate effect also observed at 48 and 72 h, respectively. Interestingly, this observation in CMJ performance was not consistent with changes in MVIC torque, however, is in line with previous studies reporting neuromuscular outcomes (MacDonald et al., 2013, 2014; Halperin et al., 2014; Jones et al., 2015; Richman et al., 2018). Evidence from other studies has demonstrated that MVIC performance is unaltered by FR (MacDonald et al., 2013; Halperin et al., 2014) and thus, suggests that FR is at least unlikely to impair the development of acute maximal strength. However, power development may be of greater importance in functional and performance tasks than maximal strength. Our findings support Pearcey et al. (2015) who suggests that FR is unlikely to benefit a single joint isometric task but rather have feasibility for multi-joint dynamic movements requiring acceleration of the body in a single plane. The reasons for this are at this stage speculative. However, our CMJ results are also in in line with the results demonstrated by MacDonald et al. (2014), who reported an increase in CMJ at 48 h following a high-volume back squat protocol, and are similar to other massage interventions (Mancinelli et al., 2006; Willems et al., 2009). Therefore, acute FR may offer task specific performance improvements. Specifically, the attenuation of power loss appears to be the most likely during recovery from damaging ECC exercise although the factors contributing to this response are yet to be fully elucidated.

The results of this study showed an increase in pain tolerance at 48 h for the FR condition, with moderate effects also observed at post-training, 24, 48 and 72 h. Several investigations have demonstrated an improved pain tolerance in the lower limbs with FR (Saxton and Donnelly, 1996; MacDonald et al., 2014; Pearcey et al., 2015). However, the physiological mechanisms responsible remain unclear. One possibility is that massage and FR increase blood flow directly to the area (Crane et al., 2012; Hofitel et al., 2016), thus acutely aiding the removal of metabolic by-products. In the latter stages of recovery, repeated exposure to manual pressure (i.e., FR) to the agonist, synergist and antagonist musculature may modulate monosynaptic group Ia muscle spindle afferent firing in response to stretch, or, alternatively downregulate pain sensitive afferent feedback caused by inflammation (Beardsley and Skarabot, 2015). Thus, it can be theorized that this may have potentially improved stretch reflex contractility and hence the power development observed in this study, however this is speculative at this stage. Another possibility is that acute FR causes a widespread modulatory response to pain. In particular, two studies, Aboodarda et al. (2015) and Cavanaugh et al. (2017b) both showed that contralateral FR improved pain tolerance in the opposite limb. Thus, our current findings and those of Aboodarda et al. (2015) and Cavanaugh et al. (2017b) suggest that neural mechanisms may, at least in part, contribute and may involve a temporary downregulation of pain sensitive afferent pathways. Further, muscle soreness is delayed following ECC contractions, despite muscle function being impaired immediately following exercise, and thus the change in pain tolerance cannot completely explain changes/reductions in neuromuscular performance (Byrne et al., 2004). Moreover, although fatigue and pain sensitive afferent feedback has been shown to acutely reduce torque of the antagonist musculature in a flexor/extensor relationship (Kennedy et al., 2013), an immediate reduction in agonistic VA was not demonstrated in our study (i.e., quadriceps). Therefore, the relationship between poorer pain threshold and performance remains somewhat unclear. As suggested by MacDonald et al. (2014), the improvement in pain tolerance and ROM during the recovery period may be due to the facilitation of connective tissue repair. However, a clear decrease in MTC, indicative of a reduction in swelling, was not demonstrated which also renders this interpretation difficult. Thus, the mechanical and perceptual improvements observed in this study are unlikely to be explained by the suggestions of MacDonald et al. (2014).

This study did not show any significant changes in any evoked responses. Although, moderate to large effects were observed for VA and PTT_time_ at 72 h the meaning of this effect at a single time point is unclear. Following exercise, VA is thought to be affected by both central and peripheral factors (Gandevia et al., 1995). Interestingly, early ECC investigations have showed mixed results regarding VA changes in the days following exercise. For example, Gibala et al. (1995) and Saxton and Donnelly (1996) showed no change in VA despite more recent studies demonstrating the ability of fatiguing exercise to reduce VA of the quadriceps musculature (Kennedy et al., 2015; Goodall et al., 2018). Conversely, a reduction in VA has been demonstrated in the days following muscle damaging exercise causing DOMS in the elbow flexors (Behm et al., 2001). However, the results of this study suggests that FR does not improve neural activation/drive are therefore, is unlikely to explain the improvement in CMJ. Furthermore, the proposed neurophysiological changes proceeding FR proposed by other authors (Beardsley and Skarabot, 2015; Aboodarda et al., 2018), suggests that any central changes may be due to autonomic process rather than the capability to voluntarily activate the musculature. Additionally, the evoked responses obtained in this study (VA, PTT and PTT_time_) are reflective of efferent pathways and no not account for potential sensory changes that may have occurred following FR. Thus, we suggest that future studies investigate acute changes in afferent pathways such as the H-reflex response which may be more sensitive to changes caused by the innervation of muscle spindles following acute FR interventions as has recently been conducted by Young et al. (2018). Alternatively, the decrease in pain threshold may cause a downregulation of group III/IV pain sensitive afferent firing. Although group III/IV afferents have been shown to decrease VA (Kennedy et al., 2013, 2015), changes are short lived. Moreover, it is not clear if the changes observed in blood flow occlusion studies where the acute increase in metabolite concentration causes sustained group III/IV afferent firing, is consistent with the pain related afferent feedback observed during the recovery (24–72 h) following muscle damaging exercise.

Although the results of this study investigated the effect of FR on recovery following ECC exercise we acknowledge several factors that may require consideration. For example, although ECC is known to cause EIMD, biochemical markers (i.e., creatine kinase) were not measured in this study. Additionally, although a repeated bout effect may also exist, protecting against EIMD from a secondary bout of ECC exercise, the randomized and counterbalanced order of the conditions, and prolonged time between conditions likely controlled for such effect. Lastly, we acknowledge that the ECC contractions performed in the leg extensors may not entirely represent the nature of muscle damage following multi-joint exercise and thus, should be considered in future research.

Collectively, the results of this investigation provide some support for the use of FR to improve jump performance, with minimal effects on other measures of recovery following muscle damaging ECC exercise. Despite no clear evidence for a neural contribution, the improvements in jump performance may at least in part, by facilitated by an increase in pain tolerance. Furthermore, the lack of improvement in maximal force suggests task specific, rather than broad functional performance improvements may be expected. These findings are likely to hold important implications in applied sports settings where lengthening muscle contractions cause muscle damage, especially when training and competition schedules do not allow for sufficient recovery.

## Ethics Statement

This study was approved by the Charles Sturt University Human Research Ethics Committee.

## Author Contributions

ED was responsible for overseeing the project including data collection, statistical analyses and manuscript preparation. CL was contributed to the data analysis and manuscript preparation. CW, SB, and MS contributed to the study design, discussion of results and manuscript preparation.

## Conflict of Interest Statement

The authors declare that the research was conducted in the absence of any commercial or financial relationships that could be construed as a potential conflict of interest.

## Abbreviations

CMJ: countermovement jump
CON: control
DOMS: delayed onset muscle soreness
ECC: eccentric
EIMD: exercise-induced muscle damage
FR: foam rolling
MTC: mid-thigh circumference
MVIC: maximal voluntary isometric contraction
PPT: pressure-pain threshold
PTT: peak twitch torque
PTT_time_: time-to-peak twitch torque
ROM: range of motion
RTD: rate of twitch torque development
VA: voluntary activation
